# Patient perceptions of advance care planning within primary care: a systematic review of facilitators and barriers

**DOI:** 10.1186/s12875-025-03028-0

**Published:** 2025-10-31

**Authors:** Elizabeth Abbey, Katy Sunderland, Matthew Cooper, Paul Taylor, Catriona R. Mayland

**Affiliations:** 1 Division of Clinical Medicine, University of Sheffield, Sheffield, UK; 2 Sheffield Teaching Hospitals NHS Foundation Trust, Sheffield, UK; 3 The Medical School, University of Sheffield, Sheffield, UK; 4 The Library, University of Sheffield, Sheffield, UK; 5 Sheffield Centre for Health and Related Research, University of Sheffield, Sheffield, UK; 6 St Luke’s Hospice, Sheffield, UK; 7 Palliative Care Unit, University of Liverpool, Liverpool, UK

**Keywords:** Primary care, Palliative care, Advance care planning, General practice, Systematic review

## Abstract

**Background:**

Advance care planning is a key aspect of palliative care and aims to establish patient preferences for future care, benefiting patients and their families. Palliative care, including advance care planning, is often provided by primary care physicians. Levels of advance care planning, however, remain low internationally. We aimed to conduct a systematic literature review to understand the barriers and facilitators encountered by patients when considering advance care planning conversations within the primary care setting.

**Methods:**

Five electronic databases (Ovid MEDLINE, PubMed, PsycINFO, CINAHL and Scopus) and grey literature were searched in April 2025. Quantitative and qualitative data were extracted and synthesised using a convergent, integrated approach. The Mixed Methods Appraisal Tool was used to assess study quality.

**Results:**

From 2495 articles, 48 studies were included. Barriers and facilitators can each be categorized into three themes, with further subthemes: 1) Professional factors, which encompassed the relationship between patient and healthcare professional, the skills and attributes of the healthcare professional, and the specific role of the healthcare professional in the advance care planning process; 2) Patient factors, including perceptions of self, family role, personal and religious views of advance care planning, and personal characteristics; 3) Features of the advance care planning conversation.

**Conclusions:**

To enhance advance care planning uptake, there should be protection of relationships between healthcare professional and patient, adequate time for face-to-face conversations, and relevant training for healthcare professionals. More widely, increasing public awareness of these topics is vital. It is essential to balance standardisation to encourage and support these conversations, whilst maintaining an individualised approach.

**Supplementary Information:**

The online version contains supplementary material available at 10.1186/s12875-025-03028-0.

## Introduction

The demand for palliative care worldwide is rising as a result of an aging population and increasing multimorbidity, and 75% of people nearing the end of life may benefit from palliative care by 2040 [1–3]. Palliative care aims to improve the quality of life of patients with a life limiting illness and support their families [4]. It adds value from early in the disease trajectory until the very end of life. One key component of good palliative care is advance care planning (ACP).

ACP has been defined as “the ability to enable individuals to define goals and preferences for future medical treatment and care, to discuss these goals and preferences with family and health-care providers, and to record and review these preferences if appropriate” [5]. ACP has been shown to improve end of life care, patient satisfaction, and reduce stress, anxiety, and depression in family members [6, 7]. The precise structure of palliative care, including ACP, and its delivery varies worldwide, as well as within countries and between conditions. Commonly, however, palliative care and ACP is provided within primary care, with support from specialist palliative care services [8].

Primary care is “the provision of integrated, accessible health care services by clinicians who are accountable for addressing a large majority of personal health care needs, developing a sustained partnership with patients, and practicing in the context of family and community” [9].General practitioners (GPs) and their equivalents internationally (for example family physicians, family practitioners, primary care physicians, internal medicine physicians, henceforth referred to as GPs) are physicians specialising in the delivery of healthcare in a primary care setting [10–12]. GPs are often considered well placed to provide palliative care due to their community focus, sustained relationships with patients, and capacity to provide home visits [13–15]. Internationally, however, levels of ACP remain low; in Canada, for example, less than 20% adults have engaged with ACP [16]and in the United Kingdom (UK) just 5% of patients acutely admitted to hospital have an Advance Care Plan accessible to the medical team [17].

Understanding the barriers to, and facilitators of, ACP from patients’ perspectives may reveal why levels of ACP remain low. Two reviews published previously found that evidence of individual patient perspectives of barriers and enablers to ACP in primary care was limited, and most often presented through the healthcare professional (HCP) as proxy [18, 19]. One review specifically recommended that future studies should seek to address this, and engage patients directly [18].

Given the increasing demand for palliative care, low levels of ACP, and the vital role of primary care in delivering these discussions, it would be helpful to generate an up-to-date picture of this topic to guide further policies and research and optimise these processes. With prior reviews calling for further research to focus on patient perspectives, an up-to-date review is warranted to assess whether this gap has been addressed. Our aim, therefore, was to conduct a systematic literature review to understand the barriers and facilitators reported by patients when considering ACP conversations within the primary care setting.

## Methods

### Literature review question

The specific question to be addressed by the systematic review was:

What are the barriers to, and facilitators of, ACP within primary care as reported by patients?

### Design

A systematic review was the chosen methodology in order to generate a comprehensive and unbiased summary of the relevant literature [20]. The review was conducted according to Joanna Briggs Institute guidance and reported in line with the Preferred Reporting Items for Systematic reviews and Meta-Analyses (PRISMA) checklist (Additional File 1) [20, 21].

### Study selection

A search strategy was developed with an experienced medical librarian (MC) and was based on terms related to the research question; “primary care”, and “advance care planning”. Search terms were broad to prevent excluding any potentially relevant articles. The electronic databases Ovid MEDLINE, PubMed, PsycInfo, CINAHL and Scopus were searched, which encompass a broad range of publications including medical, nursing and psychological standpoints. The search was limited to articles published between 2012 and 2025, journal articles, articles published in the English language, and articles relating to humans. The concept of ACP was first recognised as early as the 1970 s and has evolved over time [22, 23]. As ACP became more widely established in clinical practice, a wave of related strategies, guidelines and laws were introduced internationally in the years leading up to 2012 [23–25]. Our search dates were therefore chosen as a pragmatic range to encompass ACP evidence as it became more widely practiced in its current form. The Ovid MEDLINE search strategy is detailed as an example (Table 1). Specific inclusion and exclusion criteria were used to screen for articles (Table 2).

**Table 1:** Ovid MEDLINE search strategy

ID	Search term
1	(“primary care” OR “general practice” OR “GP” OR “general practitioner” OR “family physician” OR “family practitioner”)
2	(“advance care planning” or “advance directive” or “advance care plan” or “advance decision” or “advance statement” or “living will”)
3	1 AND 2
4	3 [DT 2012–2025] [Document type Journal Article] [Languages English] [Humans]

**Table 2:** Inclusion and exclusion criteria

Inclusion criteria
• Primary research of any design • Describes barriers to, and/or facilitators of, ACP within primary care reported by patients, nursing home residents, older adults, or the general public • Published in English language • Published between 2012 and search date • Relates to individuals > = 18 years old
Exclusion criteria
• Articles such as editorials, commentary or opinion pieces, conference abstracts, case series, case reports, and books. • Does not describe barriers to, and/or facilitators of, ACP within primary care reported by patients, nursing home residents, older adults, or the general public • Published in language other than English • Published prior to 2012 • Relates to individuals < 18 years old

*ACP* Advance care planning

Only studies relating to adults were included, recognising that the provision of palliative care and discussion of ACP with children and their parents is different to that in adults, with diverse challenges and requiring unique approaches [26, 27]. Studies including the opinions of multiple different groups, such as HCPs or family members and patients, were included only if they reported patient data separately. Similarly, studies including data on ACP in multiple or unspecified settings were included if they reported findings related to ACP in primary care separately. We included studies which recruited from settings outside of primary care (for example secondary care clinics or community events) if these papers reported findings related to ACP in primary care. We included studies which recruited people with palliative care needs, including patients, nursing home residents, and older adults. We also included studies which recruited members of the general public, acknowledging that ACP is relevant to all of these groups and that they may all have useful insights into barriers and facilitators. Henceforth we refer to all these groups as ‘patients’ for simplicity. Multiple search terms were used to capture studies relating to different forms of ACP, and the setting of primary care in different countries. We included studies which reported on ACP undertaken by any HCP within primary care. This included GPs and their equivalent role internationally (for example family physicians and primary care physicians), as well as other HCPs working within primary care, for example community nurses.

An electronic literature search was conducted on 27th April 2025. An initial title and abstract screen, followed by a full text review of any potentially eligible articles, was completed by two independent reviewers (EA and KS). Any conflicts were resolved through discussion with a third reviewer (PT). Reference lists of relevant review articles and were checked for additional relevant papers. A grey literature search was also undertaken by searching Grey Matters [28]EThOS [29]the catalogue at The British Library [30]Open Grey [31]Proquest [32]the Social Policy and Practice website [33]and search engines including Google and Google Scholar. This did not yield any papers for screening.

### Data extraction

Quantitative and qualitative data were extracted from included studies by two independent reviewers (EA and KS) using a proforma designed by the research team and piloted before use (Additional file 2). Data were mapped out in a descriptive manner according to the following: country, setting, population characteristics, aim/s, methods and findings. Extracted data were mapped to key components of the research question: barriers to ACP within primary care as reported by patients; and facilitators to ACP within primary care as reported by patients. In addition, information was collected on the study aim(s), location/setting, method and population. Triangulation was used to compare extracted data and any disagreements were resolved through discussion with a third reviewer (CRM or PT).

### Quality assessment

The Mixed Method Appraisal Tool (MMAT) was used to critically appraise the quality of included studies [34]. This tool was chosen due to the heterogeneity of included study types. Eligible studies were critically appraised by two independent reviewers (EA and KS) with a third reviewer (PT) available to assess discrepancies if needed. Results were used to inform about study quality but did not inform inclusion or exclusion of studies in the review. Studies were scored out of five according to the MMAT criteria, scoring one point for each of the MMAT criteria met. Full details of the MMAT scores can be found in Additional file 3.

### Data transformation

As the included studies were of quantitative and qualitative data, data transformation was performed to facilitate integration and synthesis. Quantitative data were converted to ‘qualitised data’, as recommended by the Joanna Briggs Institute (JBI) methodology for mixed methods systematic reviews [35]. This allows quantitative data to be interpreted alongside qualitative data to produce a consistent analysis. This process involved the transformation of numerical data into textual descriptions. For example, quantitative findings of descriptive statistics, such as percentages or frequencies, were summarised in text form. Examples of qualitised data are presented in Additional File 4. ‘Qualitisation’ was performed independently by two reviews (EA and KS) and cross-checked, with a third reviewer (PT) available to resolve any discrepancies.

### Data synthesis and integration

This review used a convergent integrated approach of data synthesis and integration [35]. Therefore ‘qualitised data’ were assembled and pooled with qualitative data. Using the JBI meta-aggregative approach for qualitative systematic reviews, pooled data were then examined by EA and categorised based on similarity and content, forming themes. This produced integrated findings which answer the research question. Following triangulation and discussion with the research team, the following final themes were agreed:

- Professional factors.
- Patient factors.
- Features of the ACP conversation.

## Results

**Fig. 1:**
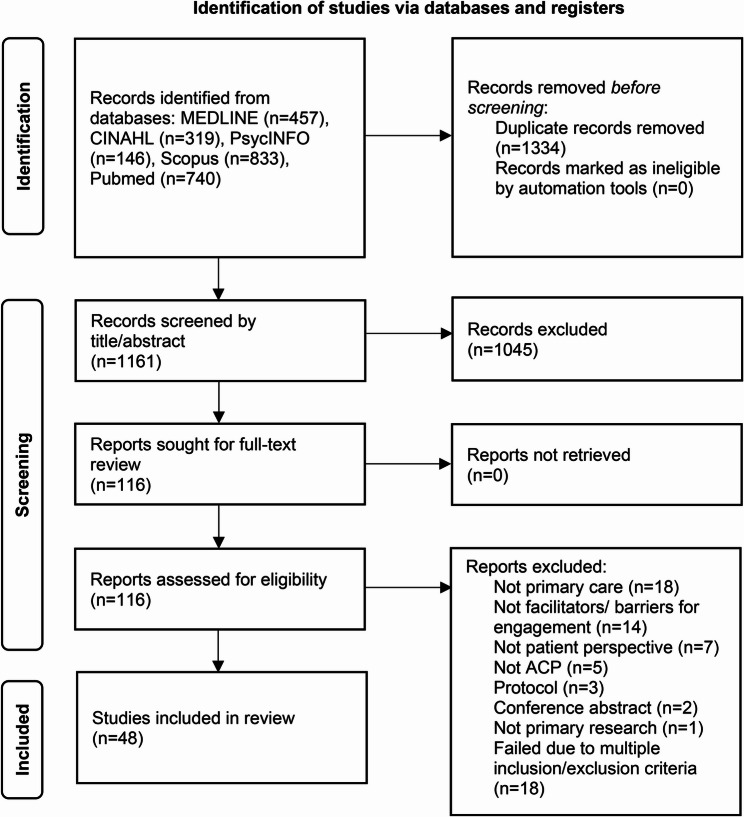
Flow diagram for systematic review process

From 2495 initial search results, 1161 were screened for eligibility, of which 48 were included in the systematic review. The screening process is outlined in Fig. 1.

### Study characteristics and quality assessment

The 48 included studies were conducted across twelve countries: the United States of America (USA) (*n* = 11) [36–46], Belgium (*n* = 7) [47–53], the UK (*n* = 7) [54–60], Japan (*n* = 6) [61–66], the Netherlands (*n* = 5) [67–71], Canada (*n* = 4) [72–75], Australia (*n* = 2) [76, 77], Norway (*n* = 2) [78, 79], Israel (*n* = 1) [80], Hungary (*n* = 1) [81], Germany (*n* = 1) [82], and Malaysia (*n* = 1) [83]. The majority of studies were qualitative in nature (*n* = 21) [36, 38, 39, 41, 42, 44, 48, 51, 52, 55–57, 68, 69, 72–74, 77–80] using interviews and focus groups to collect data. Fourteen studies used quantitative methods only (*n* = 14) [37, 46, 47, 49, 61, 63–65, 67, 75, 76, 81–83], utilising questionnaires and surveys, and the remainder used mixed methods (*n* = 13) [40, 43, 45, 47, 50, 54, 58, 59, 62, 66, 70, 71, 84].

The majority of studies recruited participants from general practice, primary care or a family practice (*n* = 26) [38–40, 42, 43, 45–48, 50–55, 59, 63, 66, 68, 74, 75, 77, 78, 82–84]. Few studies recruited from related community care settings; home medical care clinics (*n* = 1) [61], community clinics (*n* = 1) [80], and family health teams (*n* = 1) [72]. A minority recruited from hospital (*n* = 2) [58, 65], community organisations (*n* = 2) [49, 70], and unspecified ‘Health Systems” (n = 2) [36, 41]. Other recruitment settings were care homes (*n* = 2) [76, 79], a commercial research company (*n* = 1) [76], the general population (*n* = 2) [62, 81] and a combination of settings (*n* = 7) [44, 57, 64, 67, 69, 71, 73]. One study did not clearly define their recruitment setting (*n* = 1) [37].

The majority of studies discussed ACP in primary care conducted by GPs (*n* = 16) [47, 49–54, 56, 58–60, 67–70, 76] or equivalent (family doctors/physicians (*n* = 9) [45, 50, 61, 62, 64, 73–75, 80], primary care physician/doctor (*n*−12) [37–40, 42, 44, 46, 63, 65, 66, 71, 83]). One study reported on ACP completed by general practice nurses (*n* = 1) [77]. Some papers reported on ACP completion by multi-professional teams (*n* = 8) [36, 41, 55, 72, 78, 79, 81, 82], or did not specify the specific role of the HCP completing ACP (*n* = 2) [43, 57].

Papers reported barriers to, and facilitators of, ACP in primary care as perceived by patients (*n* = 23) [37, 38, 41–44, 47, 48, 53–55, 57, 58, 60, 61, 63, 64, 69, 70, 77, 78, 80, 83], older adults (*n* = 19) [36, 39, 40, 45, 46, 50–52, 59, 65–68, 71–75, 82], the general public (*n* = 4) [49, 62, 76, 81] and nursing home residents (*n* = 2) [56, 79]. Study characteristics are displayed in Table 3.

**Table 3:** Characteristics and quality appraisal of studies included in the systematic review

First author and year	Title	Aim/s	Location/setting	Method/population	MMAT score
Ferguson CM *et al*, 2024 [43]	Action plans increase advance care planning documentation and engagement among English and Spanish-speaking older adults.	To determine whether the creation and completion of an ACP-AP results in increased ACP documentation and engagement among English and Spanish-speaking older adults.	USA. Primary care clinics in San Francisco Health Network and San Francisco Veterans Affairs Medical Center.	Mixed-Methods Secondary and cross-sectional data at baseline and the six-month follow-up timepoint from two randomized controlled trials. 586 participants who were aged 55 years or older, spoke English or Spanish “well” or “very well,” had two or more chronic medical conditions, and two or more primary care visits and emergency department or hospital visits in the past year.	4
Driller B *et al,* 2024 [78]	Normality and compassionate care: experiences from advanced cancer patients in their last time at home.	To gain insights from experiences of advanced seriously ill cancer patients at home while receiving palliative treatment and being engaged in ACP within primary healthcare settings.	Norway. Primary healthcare setting in a rural area; ACP conversations offered by GPs, homecare nurses and community cancer nurses.	Qualitative. Individual, semi-structured interviews. 12 participants who had advanced non-curable cancer in a palliative setting with an estimated survival time of less than three months.	4
Stevens J *et al, *2024 [53]	Experiences with implementing advance care planning (ACP-GP) in Belgian general practice in the context of a cluster RCT: a process evaluation using the RE-AIM framework	To evaluate experiences with implementation of the intervention, as reported by patients and GPs who participated.	Belgium. Multiple general practices.	Mixed-Methods. A process evaluation following the Reach, Effectiveness, Adoption, Implementation, Maintenance (RE-AIM) framework. Data sources include recruitment and implementation monitoring, questionnaires for patients and GPs, and semi-structured (focus group) interviews with patients and GPs. 18 Belgian GPs and 53 patients with chronic, life-limiting illness (advanced/unresectable cancer, organ failure, frailty), for whom the GP would not be surprised if they were to die within the next 12–24 months.	5
Winnifrith T *et al,* 2024 [54]	Proactive advance care planning conversations in general practice: a quality improvement project	To assess the take-up rate and acceptability in general practice of a timely and personalised ACP conversation using a ‘What matters to you’ framework, and to ensure that different diagnostic and demographic groups were included.	UK. Single general practice.	Mixed-Methods. Participants were offered an ACP conversation; a survey sought feedback. 115 patients without previous ACP and potentially in the last year of life.	3
Yoshihara-Kurihara H *et al,* 2024 [65]	Effectiveness of initiating advance care planning among older outpatients through intervention by physicians trained in a model discussion video: A randomized controlled trial	To assess the impact of a physician-led intervention on ACP introduction among older outpatients using a model discussion video.	Japan. Two internal medicine outpatient departments of two secondary emergency hospitals.	Quantitative. Prospective interventional study where the primary outcome was ACP discussion occurrence. Secondary outcomes included ACP engagement, engagement score for advance directives acquisition and score for surrogate decision-maker identification, and anxiety incidence. 48 Japanese outpatients aged ≥65 years who were regular visitors of the hospital, had made multiple visits previously and were capable of attending appointments independently or with assistance.	3
Eli K* et al,* 2024 [55]	Patient and relative experiences of the ReSPECT process in the community: an interview-based study	To explore how patients and relatives in community settings experience the ReSPECT process and engage with the completed form.	England. 13 general practices.	Qualitative. Semi-structured interviews were conducted with patients, the relatives of patients who lacked capacity, and pairs of patients and relatives. 13 interviews; six with patients, four with relatives, and three with pairs of patient and relative.	5
Izumi SS* et al,* 2024 [44]	Advance care planning as perceived by marginalized populations: Willing to engage and facing obstacles	To describe how patients from marginalized populations experience and perceive ACP.	USA. Four primary care clinics and one nursing home in a Pacific Northwest city.	Qualitative. Interpretive phenomenological approach with semi-structured qualitative interviews. 30 patients from marginalized populations with serious illness.	5
Tietbohl CK *et al,* 2024 [45]	A Mixed-Methods Comparison of Interventions to Increase Advance Care Planning	To compare a passive intervention (mailed materials) to an interactive intervention (group visits) on participant ACP engagement and experiences.	USA. Primary care clinics.	Mixed-methods. Draws on interview and survey data collected for a two-arm randomized clinical trial comparing ACP group visits and mailed materials. 110 patients who were age 60 years or older and their primary care clinician determined that they were appropriate for group visits.	4
Caplan H *et al,* 2024 [46]	Assessment of Feelings Towards Advanced Care Planning in the Latino Community	To understand how conversations about ACP are perceived by Latino patients in a primary care setting.	USA. Single urban family medicine clinic.	Quantitative. Retrospective analysis of survey data. 33 patients over the age of 50 who identified as Latino and were available at the clinic on the day of survey administration.	5
Gerger H *et al,* 2024 [71]	Adjusting advance care planning to older people's needs: results from focus groups and interviews	To assess whether different types of older people can be identified according to their views and needs about the last phase of life and ACP, and how the different types of older people can be approached in an adequate way by health care professionals in ACP conversations?	The Netherlands. General practice internal networks, guided group activities for older people (e.g. coffee or a bingo afternoon), and a public regional health fair.	Mixed-methods. Questionnaire used for purposeful selection of participants. Then two focus groups and individual interviews. 15 older adults, aged 70 or older, with diverse health care needs, diverse social and cultural backgrounds, living at home.	5
Andrews N *et al, *2023 [56]	‘I don’t think they really link together, do they?’ An ethnography of multi-professional involvement in advance care planning in nursing homes	To understand what factors influence multi-professional involvement in the ACP process within nursing homes, and how multi-professional working impacts the ACP process in nursing homes?	UK. Two nursing homes.	Qualitative. Unstructured observation, formal and informal interviews and document review. Six residents, four relatives, 19 nursing home staff and seven visiting professionals from participating nursing homes were included.	5
Demirkapu H *et al,* 2023a [51]	Advance care planning among older adults in Belgium with Turkish backgrounds and palliative care needs: A qualitative interview study	To examine ACP knowledge, experiences, views, facilitators and barriers in the under-researched population of older Turkish-origin patients in Belgium requiring palliative care	Belgium. General practices in Brussels or Antwerp.	Qualitative. Semi-structured interviews. 15 older adults aged 65-89, all of whom were first-generation immigrants to Belgium and identified as Muslim.	5
Demirkapu H *et al,* 2023b [52]	Advance care planning among older adults of Moroccan origin: An interview-based study	To explore ACP related knowledge, experience, views, facilitators and barriers among older Moroccan adults in Belgium.	Belgium. General practices in Brussels or Mechelen.	Qualitative. Semi-structured interviews. 25 Belgian residents of Moroccan origin aged ≥ 65 years in primary care without life-threatening illnesses who could benefit from ACP discussions.	5
Nimmons D *et al,* 2023 [57]	Views of people living with dementia and their carers on their present and future: a qualitative study	To explore the views and perceptions of dementia and the future of people living with dementia in England, with a focus on end of life.	England. NHS memory services, general practice, carer or dementia organisations, and the NIHR Join Dementia Research website.	Qualitative. Semi-structured interviews, analysed using reflexive thematic analysis. 11 people living with dementia and six family members.	5
Stevens J *et al*, 2023 [47]	Advance care planning engagement in patients with chronic, life-limiting illness: baseline findings from a cluster-randomised controlled trial in primary care.	To assess whether patient characteristics and patient-perceived quality of ACP communication in GP were associated with engagement.	Belgium. General practices in Flanders or Brussels.	Quantitative. Written questionnaire. Baseline data from a cluster-randomised controlled trial aiming to evaluate an ACP intervention in GP. Dutch- speaking adults with a chronic, life-limiting illness for whom their GP answered ‘no’ when asked: ‘Would I be surprised if this patient were to die within the next 12 to 24 months?’	5
Hayashi S *et al,* 2023 [61]	Relationship between patient-centred care and advance care planning among home medical care patients in Japan: the Zaitaku evaluative initiatives and outcome study	To examine the association between the quality of primary care and ACP participation among patients receiving home-based medical care.	Japan. 29 home medical care clinics located in the Tokyo Metropolitan area, the Nara Prefecture, and the Nagasaki Prefecture in Japan.	Quantitative. Written questionnaire. Data collected as part of wider, multi-centre cross-sectional study. Adult Japanese patients receiving home medical care from home care physicians working at one of 29 participating clinics, who were able to respond to the survey.	3
Finkelstein A *et al*, 2023 [80]	Promoting advance care planning (ACP) in community health clinics in Israel: Perceptions of older adults with pro-ACP attitudes and their family physicians	To understand the barriers and facilitators to ACP conversations between patients and family physicians, and the signing of advance directives in community health clinics.	Israel. Two health community clinics in Jerusalem.	Qualitative. Semi structured interviews with patients, and focus groups with family physicians. 28 patients identified by their family physicians as having an interest in advance care planning, and 11 family physicians.	5
De Vleminck A *et al*, 2023 [48]	Emotional cues and concerns of patients with a life limiting, chronic illness during advance care planning conversations in general practice	To explore; to what extent patients with serious illness express emotional cues and concerns during advance care planning conversations with their GP, the content of cues/concerns, and GPs’ responses to cues/concerns.	Belgium. General practices in Flanders or Brussels.	Qualitative. Coding and thematic analysis of 20 ACP conversations. 21 patients who were Dutch- speaking adults with a chronic, life-limiting illness for whom their GP answered ‘no’ when asked: ‘Would I be surprised if this patient were to die within the next 12 to 24 months?’ and 11 GPs.	5
Bzura M *et al, *2022 [75]	Engagement and attitudes towards advanced care planning in primary care during COVID-19: A cross-sectional survey of older adults	To determine the attitudes and engagement in advance care planning according to the Stages of Change among older adults in a primary care setting and to determine the impact of COVID-19 on advance care planning.	Canada. COVID-19 vaccination event offered to patients of a large urban academic primary care outpatient clinic.	Quantitative. An anonymous, self-administered cross-sectional survey. 134 patients aged ≥70 years attending the vaccination event.	3
Smith KM *et al*, 2022 [36]	Perceived Barriers and Facilitators of Implementing a Multicomponent Intervention to Improve Communication With Older Adults With and Without Dementia (SHARING Choices) in Primary Care: A Qualitative Study	To explore barriers and facilitators to the implementation of SHARING choices – a multicomponent intervention designed to improve ACP conversations with older adults in primary care.	USA. Two health systems in Baltimore and Washington.	Qualitative. Semi-structured interviews with patients and family participants, and focus groups with staff. 30 primary care clinicians, staff, and administrators from each health system. 22 patient and family participants recruited from primary care practices. Patients were 65 years or older and regularly attended a medical visit with an adult family, friend, or unpaid caregiver.	5
McLarty S *et al*, 2022 [37]	Provider and Patient Insights Into the Cancer Care Journey	To assess physician and patient preferences for an oncologist selection tool, involvement in cancer care, value-based care, and end-of-life planning.	USA. Recruitment setting unclear.	Quantitative. Cross sectional survey. 53 primary care providers across the USA, and 112 patients with current or previous cancer across the USA who were 25 years or older, had health insurance, and were currently undergoing or had completed chemotherapy.	3
Van der Plas A *et al*, 2022 [67]	The patient’s relationship with the General Practitioner before and after Advance Care Planning: pre/post-implementation study	To examine the association between having an ACP conversation, the patients trust in the GP, and the patient feeling the GP knows him/her.	The Netherlands. Ten GP practices and two care homes.	Quantitative. Questionnaire distributed pre- and post-ACP. 458 patients aged 75 years or older.	5
Canny A *et al, *2022 [58]	Advance care planning in primary care for patients with gastrointestinal cancer: feasibility randomised trial	To assess the feasibility and acceptability to patients, carers, and GPs of a primary care ACP intervention for people with incurable oesophageal, gastric, or pancreatic cancer.	Scotland. Regional cancer centre.	Randomised controlled trial with mixed methods. Patients randomised to ACP intervention or standard care. Qualitative interviews with purposive sampling explored patient, carer, and GP experiences. 46 patients aged 18 years or older, starting palliative oncology treatment for newly diagnosed incurable pancreatic or upper gastrointestinal (oesophageal or gastric) cancer.	5
Xu L *et al,* 2022 [38]	Patient Perspectives on Serious Illness Conversations in Primary Care	To elicit patients’ perspectives on serious illness conversations conducted by primary care clinicians.	USA. Two primary care clinics in Boston.	Qualitative. Semi-structured interviews. 11 patients who were 18 years or older, English-speaking, and able to recall having had a serious illness conversation.	5
Carter C *et al*, 2022 [72]	How the more life discourse constrains end-of-life conversations in the primary care of medically frail older adults: A critical ethnography	To understand the socio-political forces shaping EOL conversations between clinicians, medically frail older adults and/or their care partners within an urban primary care setting.	Canada. Single urban Family Health Team in Ontario.	Qualitative. Observation of practice in the Family Health Team, and interviews with clinicians, patients and care partners. 20 clinicians, 11 medically frail older adults, four care partners.	5
Gardener AC *et al*, 2022 [84]	‘I’m fine!’: Assertions of lack of support need among patients with chronic obstructive pulmonary disease: A mixed-methods study	To understand how people with COPD deny their support needs and the impact on care.	England. Primary care settings in the East of England.	Mixed-methods. Sub-analysis of existing data collected within existing study programme; identification of cases of disavowal of support needs, qualitative analysis of patient interviews, analysis of linked quantitative questionnaire data; and focus groups with healthcare practitioners in primary care. 235 patients with COPD from primary care settings in the East of England, and nine health care professionals with experience working with patients with COPD in primary care.	5
Whyte S *et al*, 2022 [76]	Cognitive and behavioural bias in advance care planning	To explore cognitive biases and key differences in communication, preference and decision-making in the context of ACP for both the general public, as well as GPs and nurses with an interest in primary care. To explore individuals’ perceptions of their role in choice and potential shared decision-making with medical experts, and how this might influence motivating engagement in ACP.	Australia. Participants recruited via commercial research company, and via healthcare conference.	Quantitative. Questionnaire. 1253 members of the general public and 117 healthcare professionals (GPs and nurses).	2
Ohnuki Y *et al*, 2022 [62]	Possible Significance of a Café-style Event to Introduce Advance Care Planning for General Citizens	To determine the impact of a café-style event to raise awareness of ACP on implementation of ACP after the event.	Japan. Participants recruited via notice in regional newspaper	Mixed methods. Questionnaire distributed after event. 14 members of the general public attended the event, and eight completed the post-event questionnaire.	3
Busa C *et al*, 2022 [81]	Who should talk with patients about their end-of-life care wishes? A nationwide survey of the Hungarian population	To explore the needs and opportunities of the general population to communicate their end-of-life care wishes and to investigate what roles are assigned to healthcare providers and family members in end-of-life care discussions.	Hungary. Nationwide survey of Hungarian general population.	Quantitative. Questionnaire. 1100 members of the public randomly sampled by geographical region and household.	4
Glaudemans J *et al*, 2020 [68]	Preventing unwanted situations and gaining trust: a qualitative study of older people and families' experiences with advance care planning in the daily practice of primary care	To explore older people’s and their families’ experiences with ACP in primary care.	The Netherlands. General practices in The Netherlands.	Qualitative. Semi-structured interviews. 22 older people with experience in ACP, and eight family members.	5
Kendell C *et al*, 2020 [73]	Patient and caregiver perspectives on early identification for advance care planning in primary healthcare settings	To examine patient and caregiver views on; practice level identification of individuals at risk of deteriorating health or dying; the use of an EMR- based algorithm for early identification in PHC settings; and preferences and challenges for ACP in PHC settings.	Canada. Primary healthcare practice and orthopaedic surgery follow-up clinic in Nova Scotia. Seniors housing complex, seniors living centre, and the community in Ontario.	Qualitative. Semi-structured interviews. 14 individuals aged 65 and older with declining health, and caregivers of individuals aged 65 and older with declining health.	5
Bernard C *et al*, 2020 [74]	Exploring patient-reported barriers to advance care planning in family practice	To understand the barriers faced by older patients regarding talking to their family members and family physicians about ACP.	Canada. 20 family practices; 13 from Ontario, five from Alberta, and two from British Columbia.	Qualitative. Questionnaire. 102 adults aged 50 years or older, able to understand English, and did not have a cognitive impairment.	5
Suen L *et al*, 2020 [42]	Thinking Outside the Visit: Primary Care Patient Perspectives on Helpful Advance Care Planning Methods	To explore patients’ perceptions and acceptability of ACP outreach methods.	USA. Urban, academic adult primary care clinic in San Francisco.	Qualitative. Semi-structured focus groups. 14 primary care patients.	4
Tsuda S *et al, *2020 [66]	Group-based educational intervention for advance care planning in primary care: A quasi-experimental study in Japan	To determine whether a video-supported group-format ACP program resulted in a better AD completion rate and a greater likelihood of familial discussion about ACP compared to an individua session with a physician; and to examine factors that affected decision among group-format participants about whether to engage in familial discussion on ACP and to write ADs.	Japan. Rural family medicine clinic.	Mixed-Methods. Quasi-experimental clinical trial in which quantitative survey data compared the effectiveness of the two interventions and qualitative data were collected from the group discussions to inform a deeper understanding of the participants' perception of ACP. 109 adults aged 65 years or older who regularly visited a PCP in the clinic for chronic illness care, had seen the PCP more than three times, and were legally competent.	5
Abu Al Hamayel *N et al*, 2019 [39]	Preparing Older Patients With Serious Illness for Advance Care Planning Discussions in Primary Care	To explore older patients’ perspectives and experiences on ACP discussions with family members and/or primary care clinicians.	USA. Suburban academic primary care clinic.	Qualitative. Semi-structured interviews. 20 patients aged 60 or older, who did not have an advance directive or similar documentation, and had a scheduled visit with their primary care clinician.	5
Miller H *et al*, 2019 [77]	Patient experiences of nurse-facilitated advance care planning in a general practice setting: a qualitative study	To explore patients' perspectives of an ACP intervention designed to address common barriers to uptake in the general practice settings.	Australia. Four general practices in Eastern Sydney.	Qualitative. Semi-structured interviews. 13 patients who had attended at least one ACP conversation with a General Practice Nurse as part of an ACP intervention.	5
Tilburgs B *et al*, 2018 [69]	The importance of trust-based relations and a holistic approach in advance care planning with people with dementia in primary care: a qualitative study	To explore barriers and facilitators for ACP with community-dwelling people with dementia.	The Netherlands. Primary and community care.	Qualitative. Semi-structured interviews. 10 people with dementia and their family caregivers, recruited during community meetings.	4
Scholten *G et al,* 2018 [49]	Advance directive: does the GP know and address what the patient wants? Advance directive in primary care	To map barriers identified by GPs and patients in preparing and discussing an advance directive.	Belgium. Community (public areas, patient platforms and senior organisations)	Quantitative. Questionnaire. 502 adults aged over 64, recruited in public areas, by electronic survey, on patient platforms and via senior organisations. Study included 502 participants.	4
De Vleminck A *et al*, 2018 [50]	Do non-terminally ill adults want to discuss the end of life with their family physician? An explorative mixed-method study on patients' preferences and family physicians' views in Belgium	To describe to what extent patients aged 50 and older who are relatively stable or in good health are thinking about the EOL and willing to discuss this with their FP, and to explore whether patients and FPs indicate the same topics as triggers for ACP discussions in family practice.	Belgium. Two rural family group practices.	Mixed-methods. Questionnaire and semi-structured interviews. 286 patients aged ≥50 years in family practice completed the questionnaire. Five patients completed interviews.	5
Lum H *et al*, 2017 [40]	A Group Visit Initiative Improves Advance Care Planning Documentation among Older Adults in Primary Care	To understand the feasibility, acceptability, and reproducibility of the initiative, and to describe reasons why patients chose to participate in this intervention.	USA. Three primary care clinics at University of Colorado Hospital.	Mixed methods. Transcript analysis from ACP group visits and review of ACP documentation. 118 patients aged 65 years or over.	3
Luck T et al, 2017 [82]	Advance directives and power of attorney for health care in the oldest-old - results of the AgeQualiDe study	To provide information on the frequency of ADs/POA in oldest-old individuals and factors associated with having completed ADs/POA.	Germany. GPs in collaboration with six study centres (Hamburg, Bonn, Düsseldorf, Leipzig, Mannheim and Munich).	Quantitative. Structured interview. 704 patients identified by their GP who were aged 75 years or older, dementia-free and had at least one contact with the GP in the prior 12 months.	5
Aoki T *et al*, 2017 [63]	Patient experience of primary care and advance care planning: a multicentre cross-sectional study in Japan	To investigate the relationship between patient experience of primary care and ACP.	Japan. 28 Primary Care Clinics.	Quantitative. Self-administered questionnaire. 535 primary care patients aged 20 years or above who visited one of the participating clinics within a one week survey period.	3
Musa I *et al*, 2015 [59]	A survey of older peoples' attitudes towards advance care planning	To assess the attitudes of older people in the East Midlands through the development and administration of a survey.	UK 13 general practices from Leicestershire and Nottinghamshire.	Mixed-Methods. Focus group and questionnaire. 1823 community dwelling older adults aged 65 or older completed the survey, and unspecified number participated in focus groups.	3
Van Wijmen M *et al*, 2014 [70]	Motivations, aims and communication around advance directives: a mixed-methods study into the perspective of their owners and the influence of a current illness	To establish what are motivations of owners of an AD to draft an AD, what do they aim for with their AD and do they communicate about their AD?	The Netherlands. Community associations (‘Right to Die-NL’ and Dutch Patient Association).	Mixed-methods. Questionnaires and semi-structured interviews. 5768 participants with advance directives completed a written questionnaire, 29 patients suffering from a chronic illness completed an interview.	4
Lim MK *et al*, 2022 [83]	*Knowledge, attitude and practice of community-dwelling adults regarding advance care planning in Malaysia: a cross-sectional study*	To assess the knowledge, attitude and practice among community dwelling adults in Malaysia regarding ACP, and its associated factors.	Malaysia. Primary care clinic at University Malaya Medical Centre, Kuala Lumpur.	Quantitative. Questionnaire. 385 community-dwelling adults (ambulatory care patients or their accompanying persons) aged 21 years old or over.	5
Reich A *et al*, 2019 [41]	Is This ACP? A Focus Group Study of Patient Experiences of Advance Care Planning	To examine patient perceptions of ACP from a geographically diverse Medicare population to better capture the typical patient population in primary care and geriatric practices across the USA.	USA. Five US Health Systems (academic, public and non-profit).	Qualitative. Focus groups. 34 Medicare beneficiaries who had engaged in or were billed for ACP.	5
Hamada S *et al*, 2019 [64]	Associated factors for discussing advance directives with family physicians by noncancer outpatients in Japan	To identify the factors associated with discussing AD by noncancer patients with their physicians.	Japan. Outpatient section of the General Internal Medicine/Family Medicine department at a small hospital or clinic in a primary care setting.	Quantitative. Cross-sectional study using a self-completed questionnaire. 336 noncancer patients aged 20 years or older who visited the site for at least six months.	5
Bollig G *et al*, 2016 [79]	They know!-Do they? A qualitative study of residents and relatives views on advance care planning, end-of-life care, and decision-making in nursing homes	To study the views of cognitively able residents and relatives on ACP, end-of-life care, and decision-making in nursing homes.	Norway. Nine nursing homes.	Qualitative. Open-ended interviews. 25 residents and 18 relatives recruited by nursing home staff.	5

Terminology and abbreviations are as used in the original paper

*ACP* advance care planning, *AD* advance directiv, *COPD* chronic obstructive pulmonary disease, *EMR* electronic medical record, *EOL* end of life, *FP* family physician, *GP* general practice, *GPs* general practitioners, *NIHR* National Institute for Health Research, *PCP* Primary Care Provider, *PHC* primary healthcare; Treatment, *POA* power of attorney, *ReSPECT* Recommended Summary Plan for Emergency Care and Treatment, *UK* United Kingdom, *USA* United States of America

### Barriers to, and facilitators of, ACP in primary care

The barriers to, and facilitators of, ACP in primary care reported by the included studies can each be categorised into three themes; professional factors, patient factors, and features of the ACP conversation. These are reported in full in Table 4. Example excerpts of raw qualitative data mapped to themes are displayed in Additional File 5.

**Table 4:** Barriers and facilitators to advance care planning in general practice, as perceived by patients

	Barriers	Facilitators
Professional factors	*Relationship with HCP* • Superficial relationship with the HCP [41, 44] • HCPs frequently changing, therefore no opportunity to develop trust [44, 70] • Patient fears ACP conversations will negatively impacting the patient-physician relationship [74] • Lack of trust in HCP [44, 68, 81] • Fear the HCP will not respect their wishes and act in their best interests in the future [41, 44, 59, 70, 80] *HCP skills and attributes* • Perceived as lacking knowledge [68, 79] • Perceived as lacking time [68] • Feel the HCP will not listen [62] • Poor communication skills of HCP [41] • Perceived as poor understanding of patients’ goals and cultural values [44] • Bias, stereotyping and poor understanding of patients’ situation [44] *Role of HCP in ACP* • Unclear explanation of ACP and its value [68, 70] • Patients trust HCPs to make right decisions for them [59, 79, 82]	*Relationship with HCP* • Close relationship with HCP, particularly over time [38, 41, 44, 46, 53, 56, 63, 64, 69, 73] • Regular contact with the HCP and primary care practice [69] • Higher overall satisfaction with the HCP (indicated by JPCAT-SF score) [61] • After ACP discussions, trust in the GP with regard to end of life care improves [38, 67, 68] *HCP skills and attributes* • Good communication skills [36, 41] • Display empathy and tolerance [36, 78] • Provides emotional support: feeling listened to, that values and preferences were being respected, that their input was valued, and that the physician was acting in their best interests [44, 73] *Role of HCP in ACP* • Clear explanation of ACP and its value [41, 54, 68] • HCP initiating and leading ACP conversation [39, 42, 45, 52, 73, 74, 76, 80, 81] • Patients and HCP equally responsible for initiating the conversation [65]
Patient factors	*Perceptions of self* • Good QOL, positive view of current health, feel too young to discuss ACP [43, 52, 53, 68, 71, 74] • Disavowal of health needs [84] • Difficulty imagining losing capacity, and the need for ACP [59, 70] • Other priorities; other health concerns, family illness [43, 45] • “Mood” (no further details given) [75] *Role of family* • Difficult family dynamics regarding ACP conversations [39, 55, 66, 73, 74] • Too emotional to discuss with family, particularly children [39, 52] • Trust relatives to make right decisions for them [43, 44, 51, 52, 57, 59, 61, 62, 79, 82] • “Family considerations” (no further details given) [75] *Personal goals and preferences* • Choose not to think ahead, prefer a ‘day-to-day’ approach [45, 51, 54, 57, 58, 79, 84] • Preference not to think about death [64, 84] • Desire to hold onto life, at any cost [72, 80] *Personal views of ACP* • Topic perceived as negative (difficult, distressing, sensitive, scary, emotional, overwhelming) and therefore avoided [50, 59, 73, 74, 84] • ACP not a priority, not enough time to do it [74, 82] • Worry their preferences will change/hard to make concrete decisions [50, 74, 80] • Worry they will make the wrong decision [51] • “I don’t want to talk about it” [61] • “I don’t feel the need to talk about it” [61, 62] • Lack of knowledge about ACP [49, 61, 62, 74, 80, 82] • ACP seen as taboo [52] *Religious influence* • Religion/culture does not allow ACP [49, 59] • Death in ‘God’s hands’, ACP won’t change what happens [59] • “Religious reasons” (no further detail given) [75]	*Perceptions of self* • Poor QOL, negative view of current health, awareness of own aging [39, 53, 68, 70, 81] • Concern about own quality of life in the future [50, 64, 79] *Role of family* • Support from family, family presence in ACP conversations [37, 41, 68, 73] • Desire to avoid burdening the family [50, 80] • Concern family decisions may not align with own wishes [50] • Experience of watching a loved one with a serious illness or receiving end of life care [50, 70, 80] *Personal goals and preferences* • Prefer to focus on quality of life over quantity [80] • Want to avoid suffering at EOL [50, 80] • Want to maintain autonomy [50, 80] *Personal views of ACP* • Publicity to normalise ACP e.g. through community centres and charities [36, 80] • No perceived disadvantages; “It can’t hurt” [53] *Personal characteristics* • Patient age around the ‘ideal’ age for ACP discussion (57–59 years) [76] • Educated to University level [81] • Lower age [81] • Patient background in medicine or law [39]
Features of the ACP conversation	• Poor explanation of ACP [46] • Difficulty navigating written forms relating to ACP [36, 62, 80] • Education about purpose of ACP did not prevent negative views [58] • Not enough time with GP in appointments [36, 39, 59, 61, 69, 73, 74, 79]	• Clar explanation of ACP [45, 51] • Face-to-face visit dedicated to ACP [73] • Reminders over time to consider ACP [42] • Written information pre- and post- discussion [36, 39, 41] • Having the conversations at home [69] • Protected time for detailed conversation – within each discussion, and through follow-up visits [38] • Embed ACP into routine care [36] • Time to prepare/opportunity to consider preferences, before discussing these with GP and family [36, 38, 39] • Correct timing of the conversations (may be early or later, depending on the individual) [69, 76] • Discussion with GP or another staff member – whoever had more time [36] • Discussion of both medical and non-medical issues [69] • Desire to make decisions themselves, without family input [80] • Agenda for the conversation, to guide discussion [36, 38] • Individualised approach [38, 73] • Opportunities for questions [51] • Clear documentation and information sharing [44] • ReSPECT form/process [55] • Wishes documented and communicated with family [53] • Opportunities to revisit discussions as health changes [53]

*ACP* advance care planning, *EOL* End of life, *HCP* Healthcare professional, *GP* general practitioner, *JPCAT-SF* Japanese version of Primary Care Assessment Tool – Short Form, *QOL* quality of life, *ReSPECT* Recommended Summary Plan for Emergency Care & Treatment

### Professional factors

#### Relationship with the HCP

A superficial relationship between HCP and patient, sometimes due to frequent changes in staff, was a barrier to ACP, whereas close relationships maintained over time enabled these conversations [38, 41, 44, 56, 63, 64, 69, 70, 73]. Some patients reported a lack of trust in the HCP delivering ACP, particularly fear that they would not act in their best interests, and this acted as a barrier to engagement [41, 59, 70, 80]. In contrast, patients from marginalised populations, including those from ethnic minority backgrounds, of lower socioeconomic status, and from the LGBTQ + community, particularly worried that HCPs would not understand their goals or cultural values, motivating them to document their wishes clearly through ACP [44]. Patients also feared that ACP conversations would damage their relationship with the HCP, particularly when this was a GP [41, 59, 70, 80]. Overall patient experience of their primary care, as scored by the Japanese version of the Primary Care Assessment Tool-Short Form (JPCAT-SF), was associated with increased chance of engaging in ACP [63].

#### HCP skills and attributes

Patient perception that a HCP was lacking in palliative care knowledge or communication skills was one barrier to ACP [41, 68, 69]. Patients felt more comfortable having these discussions with HCPs perceived as possessing good communication and listening skills, and where they provided emotional support [41, 44, 73]. A compassionate, respectful and empathetic approach from HCPs was particularly important [78]. Patients from marginalised populations reported HCP behaviours reflecting bias, stereotyping or poor understanding of patient’s individual circumstances, which discouraged engagement in ACP [44].

#### The role of the HCP in ACP

Clear explanations of ACP and its purpose encouraged engagement [41, 68]. Patients also preferred HCPs to initiate the conversation, rather than waiting for the patient to do so [39, 42, 74]. Physicians were sometimes identified as the right person to initiate ACP, for example compared to other HCPs, religious officials, or the patient themselves [46]. One study found that physicians and patients were seen as equally responsible for initiating ACP conversations [75]. Patients’ strong trust specifically in their own GP meant they sometimes felt ACP was unnecessary, as they could always rely on their GP to make the right decisions on their behalf at the time [59, 71, 79, 82].

### Patient factors

#### Perceptions of self

When patients considered themselves to be in a good state of health with a good quality of life they were less likely to take part in ACP conversations, compared to those with poorer health and worse quality of life [39, 43, 52, 53, 68, 70, 71, 74]. Some patients found it difficult to imagine losing capacity, and therefore the need for ACP [59, 70]. In contrast, others were concerned for their quality of life in the future, and wanted to prepare for this [54, 64, 79].

#### Role of family

Patients described family support and their presence in ACP as key to engaging in these discussions [41, 52, 71, 73]. In contrast, several studies demonstrated that family involvement could be a barrier, due to disagreements, difficult dynamics, and the emotional distress of discussing this topic with family, particularly children [39, 52, 66, 73, 74, 78]. Past experiences of seeing relatives or friends with illness, or receiving end of life care, encouraged patients to plan for their own future [44, 51, 70]. Some patients felt no need to engage with ACP, as they trusted their family to make the right decisions for them [43, 44, 51, 52, 57, 59, 71, 79, 82].

#### Personal views of ACP

Some patients preferred to live ‘day-by-day’, avoiding thoughts of the future and in particular of death [45, 51, 54, 57, 64, 79]. Several studies reported that a barrier to ACP conversations was patient perception of the topic as too difficult, emotional and frightening [53, 54, 59, 73, 74, 78]. Some patients viewed ACP as low priority in their busy lives [74, 82]. Others feared they would make the wrong decision [51]or that their preferences would change over time [74]. Knowledge of ACP prompted engagement, whereas some patients had limited awareness of ACP and thus had not considered it or did not know how to pursue it [44, 45, 47, 49, 52, 65, 74, 82]. For some, ACP was a taboo subject which could not be discussed freely [52]. Media messaging around ACP was seen to normalise the subject and encourage engagement [39, 47, 80].

#### Religious influence

Some religious beliefs were a barrier to ACP, for example the belief that death is in ‘God’s hands’ and ACP is therefore futile [59]. Some individuals felt that ACP was not permitted in their religion [49, 59].

#### Personal characteristics

Although one study did not find any specific demographic or clinical characteristics were associated with ACP engagement, others reported that those educated to university level, and with a background in medicine or law were more likely to participate in ACP [39, 81]. One study found a patient age of 57–59 years the ‘ideal’ age to initiate ACP [76].

### Features of the ACP conversation

The way in which ACP was undertaken was key to participation; dedicated GP visits at home [69, 73]regular reminders [42]and the provision of written information were all beneficial [39, 41]. Other facilitators included the opportunity to prepare for the conversation beforehand [39]opportunities for questions [51]and discussion of both medical and non-medical matters [69]. Timing the conversation correctly was important, with some patients preferring the discussion soon after a new diagnosis of an incurable illness, however this was not unanimous [69]. Patients valued the opportunity to revisit discussions at intervals as their health changed [53]. Patients reported that the ReSPECT form (a summary of personalised recommendations for an individual’s clinical care in a future emergency in which they cannot express their wishes) [85] guided decision making and enabled patient’s wishes to be conveyed to HCPs [55]. Clear explanations of end of life care and ACP concepts enhanced engagement, although this was not always provided [45, 46, 51]. It was important that patients felt supported emotionally and that the approach was individualised to each patient [73]. Clear documentation and information sharing was an important facilitator, without which ACP information was often lost and patients had to repeat conversations on multiple occasions to different HCPs, eroding trust and patients’ willingness to engage [44]. Multiple studies reported that limited time with HCPs, particularly GPs, was a barrier to satisfactory ACP [39, 44, 59, 69, 74, 75, 79].

## Discussion

### Main findings

This review highlights the breadth of factors influencing ACP completion in primary care. The HCP has a pivotal role, both in terms of individual skill and attitude, and also working within the wider setting of primary care as a whole. Systems level constraints, such as limited time and continuity of care, are key challenges. Characteristics of the individual patient, including their perception of their own health, goals and their family relationships, also impact engagement. This variability supports the need for an individualized approach. Alongside a flexible approach, however, specific features of the ACP conversation have been identified which enable effective discussion. Conversations which are time-protected, occur face-to-face at the patient’s home, and are with a known and trusted HCP, facilitate engagement with ACP. Provision of written information ahead of time, and after the discussion, is also appreciated by patients.

### What this study adds

Palliative care is concerned with improving the quality of life for patients facing life-threatening illness and their families, including through the delivery of ACP, frequently in primary care settings [4, 86]. It is important, therefore, that the delivery of ACP is viewed in the wider context of the community healthcare systems. This systematic review builds on previous reviews of barriers and facilitators to ACP in primary care, which identified a paucity of evidence on the perceptions of patients themselves.

This review provides an up-to-date picture of our knowledge in this area. Increasing numbers of relevant studies over recent years reflects an effort to fill this gap, the recognition of ACP as an important topic internationally, and the rising role of primary care in delivering these discussions. It also reinforces value of this review as an up-to-date summary of a rapidly growing area of research.

Significant changes are being seen in the delivery of primary care internationally; workload is increasing whilst numbers of full-time equivalent practicing GPs are falling [87–95]. Major restructuring is now being seen within healthcare systems, for example through the development of Primary Care Networks in the UK, where groups of general practices work together to provide integrated services to larger populations [96]. There is also increasing employment of allied health professionals, such as physicians’ associates, worldwide, including in Australia, the Netherlands, Germany, India and Canada [95, 97]. One recognised effect of this new landscape of community healthcare has been a reduction in continuity of care for patients, who are less likely to have the majority of their care provided by a single named GP [95]. Patients report that continuity facilitates safe care, consistent advice, trust and respect between them and their physician [98]. When continuity is lacking, patients may feel that they are not taken seriously or believed by their GP [98]. In our review, a close and trusting relationship with a HCP with whom they had regular contact was a key facilitator of ACP for patients. It is striking that the way in which primary care is currently evolving may limit opportunities for these positive relationships. Systems-level changes could address this, as has been acknowledged; there have been recent calls from within the specialty for a renewed focus on continuity of care with GPs, for example [99]. It is important to note, however, that whilst most evidence we found related to relationships between patients and GPs, there was evidence that ACP was enabled through continuity of care between patient and HCP, whether or not that HCP was a GP or in another role. This should prompt further consideration of the role of these other HCPs in ACP in more detail, as well as the protection of their continuity of care for patients seeing others HCPs, particularly where they are conducting these conversations.

A panel of international experts have previously encouraged the initiation of ACP by non-physicians [5]. In hospital and community settings, including the emergency department, nurses have reported feeling well placed to conduct ACP due to their time at the bedside and strong communication skills [100–102]. In the community, a structured, nurse-led ACP intervention post-discharge from hospital has been shown to improve ACP completion and clarity of patient’s wishes [103]. We found relatively few studies which reported on ACP conducted by these HCPs, although the majority of patient-perceived barriers and facilitators focussed on factors such as trust and prior relationship with the HCP, skill and knowledge of the HCP, and the time available for the conversation, rather than the specific job-role of the HCP. Whilst further evidence on ACP provided by non-physicians is warranted, harnessing the skills and expertise of non-physician HCPs, who are increasingly present in the primary care workforce, seems a sensible approach.

An additional change seen recently within primary care which is likely to impact the delivery of quality care is the increase in time pressure, with short appointment times plus the rising use of phone and online consultation methods, accelerated by the COVID-19 pandemic [104–106]. In our review, inadequate time in appointments was a frequently cited barrier to ACP by patients, whereas face-to-face visits, particularly at home, were a facilitator to engagement. The importance of this has recently been recognised in the UK by the Royal College of Physicians. They have recommended an increase in standard appointment length from 10 to 15 min, and longer for particularly complex cases [107]. A related factor is the use of different consultation techniques. Online and telephone consultations may increase efficiency and timely access to care [106]but face-to-face consultations are preferred by patients when discussing sensitive or complex topics and so should remain the gold standard for ACP [108].

Our review highlighted the importance of the input of the individual HCP in the ACP conversation. Patients were less likely to engage in ACP when they believed that their HCP was lacking in knowledge and communication skills. Previous studies have shown that whilst GPs themselves generally have positive attitudes towards ACP, they also report a lack of knowledge, confidence and skills in this area, and would welcome further training to address this [109–112]. Indeed, targeted ACP training for GPs has been associated with improved readiness to deliver ACP in terms of willingness and confidence, and nurses asked about delivering ACP have cited increased education and support in this area as key to successful delivery [113].

It follows that with improved training HCPs in primary care may be more likely to initiate ACP, rather than waiting for the patient to do so. Having a HCP initiate and lead the ACP conversation was a frequently cited facilitator of ACP in our review. This may be linked to our findings that patients avoid ACP conversations, in particular initiating them, due to the topic feeling too challenging, emotional or scary, or due to lack of knowledge about the concept. Death and dying are well-recognised as taboo subjects in many societies [114]. GPs have previously been well-placed to address this, as part of a wider public health approach to palliative care encompassing interventions such as ‘death cafes’ and ‘compassionate communities’ [115]. Furthermore a previous systematic review found that mediated ACP interventions, such as media, print and mass-media public health awareness campaigns, are useful tools to encourage ACP in adults [116].Our findings add weight to the potential benefit of these approaches to combat the taboo and raise public awareness of the importance of ACP.

When considering the specific structure of ACP conversations, our review highlighted several features which may facilitate engagement. The provision of written information was helpful to patients, as was prior knowledge that the conversation was going to take place, to allow them to consider their preferences before discussing them with the GP and family. This is in agreement with a previous systematic review of community-based ACP interventions [117]. The development of standardised tools to aid these conversations and provide patients with written information may help to improve ACP in primary care and may also increase HCP confidence in these complex consultations. Yet patients preferred ACP which was personal to them, and had different opinions on features of ACP, such as the best time to approach it. Therefore the need for standardisation must be carefully balanced with patients’ individual circumstances and preferences.

There was some evidence that personal characteristics of the patient, such as age, profession, and family history of terminal illness may influence their engagement with ACP in primary care. Whilst it is difficult to address these, they represent populations who may benefit from targeted encouragement and education about ACP, in order to maximise uptake in individuals who may benefit.

### Strengths and limitations of the study

The use of a mixed-methods systematic review is a key strength of this review, enabling integration of results and a deeper understanding of patient experiences of ACP in general practice. We utilised the MMAT for quality appraisal of studies, which has been shown to be a reliable and valid assessment tool. We did not use the MMAT results to interpret the findings, however we have reported our interpretation transparently and made MMAT results available to support readers’ interpretation of the findings. The review was undertaken by two independent reviewers, with discussion with a third reviewer used to resolve any disagreements. The review included studies undertaken in a variety of different countries across different continents. Whilst this demonstrates the importance of this research topic internationally, the results must be interpreted in the context of varying healthcare models, both within general practice and wider healthcare delivery. The included studies comprised descriptive, quantitative non-randomised and qualitative studies, designs often associated with lower quality evidence.

## Conclusion

Understanding the barriers to, and facilitators of, ACP in primary care is important to enhance care offered to patients and to effectively target future approaches and policies in this area. Within primary care delivery, the protection of HCP-patient relationships, embracing the role of non-physician HCPs, improving relevant training for HCPs, and encouraging face-to-face conversations with adequate time may all enhance the uptake and benefits of ACP. In a wider context, ongoing efforts to break down societal taboos around death and dying are valuable, increasing the awareness and acceptance of these topics by the public. Striking the balance between standardised tools to support these conversations, whilst maintaining an individualised approach, is also useful.

## Supplementary Information

Additional file 1.

Additional file 2.

Additional file 3.

Additional file 4.

Additional file 5.

## Data Availability

The datasets used and/or analysed are available from the corresponding author on reasonable request.

## Abbreviations

ACP: Advance care planning
AD: Advance directive
COPD: Chronic obstructive pulmonary disease
EMR: Electronic medical record
EOL: End of life
FP: Family physician
GP: General practitioner/general practice
HCP: Healthcare professional
JPCAT-SF: Japanese version of Primary Care Assessment Tool – Short Form
MMAT: Mixed Methods Appraisal Tool
NIHR: National Institute for Health and Care Research
PCP: Primary care provider
PHC: Primary healthcare
POA: Power of attorney
PRISMA: Preferred Reporting Items for Systematic reviews and Meta-Analyses
ReSPECT: Recommended Summary Plan for Emergency Care and Treatment
QOL: Quality of life
UK: United Kingdom
USA: United States of America
