# Supplementary material for: Patient perceptions of advance care planning within primary care: a systematic review of facilitators and barriers
Source: BMC Prim Care. 2025 Oct 31;26:337. doi: 10.1186/s12875-025-03028-0 (PMC12577347; doi:10.1186/s12875-025-03028-0)
Supplement: Supplementary file 2 — Additional file 2. [file 12875_2025_3028_MOESM2_ESM.docx]

**Additional file 2** Data extraction form

|  | **Results** |
| --- | --- |
| **Title** |  |
| **Author** |  |
| **Year of publication** |  |
| **Country of origin** |  |
| **Citation** |  |
| **Setting** |  |
| **Aims / purpose** |  |
|  |  |
| **Study population and size** |  |
|  |  |
| **Type of study / methods** |  |
|  |  |
| **Key findings that relate to review question**  What are the barriers and facilitators to ACP within primary care as reported by patients? |  |
| **Specific sub-question** | **Findings** |
| 1. Barriers |  |
| 1. Facilitators |  |

Issues for further discussion:

Additional comments:

Reference list:
