# Supplementary material for: Patient perceptions of advance care planning within primary care: a systematic review of facilitators and barriers
Source: BMC Prim Care. 2025 Oct 31;26:337. doi: 10.1186/s12875-025-03028-0 (PMC12577347; doi:10.1186/s12875-025-03028-0)
Supplement: Supplementary file 3 — Additional file 3. [file 12875_2025_3028_MOESM3_ESM.docx]

**Additional file 3** MMAT scores for included articles, as agreed by reviewers.

| **First author and year** | **Screening questions** | | **Qualitative studies** | | | | |
| --- | --- | --- | --- | --- | --- | --- | --- |
|  | **S1. Are there clear research questions?** | **S2. Do the collected data allow to address the research questions?** | **1.1. Is the qualitative approach appropriate to answer the research question?** | **1.2. Are the qualitative data collection methods adequate to address the research question?** | **1.3. Are the findings adequately derived from the data?** | **1.4. Is the interpretation of results sufficiently substantiated by data?** | **1.5. Is there coherence between qualitative data sources, collection, analysis and interpretation?** |
| Driller B *et al*, 2024 | Yes | Yes | Yes | Yes | Can’t tell | Yes | Yes |
| Eli K *et al*, 2024 | Yes | Yes | Yes | Yes | Yes | Yes | Yes |
| Izumi S *et al*, 2024 | Yes | Yes | Yes | Yes | Yes | Yes | Yes |
| Gerger H *et al*, 2024 | Yes | Yes | Yes | Yes | Yes | Yes | Yes |
| Andrews N *et al*, 2023 | Yes | Yes | Yes | Yes | Yes | Yes | Yes |
| Demirkapu H *et al*, 2023 | Yes | Yes | Yes | Yes | Yes | Yes | Yes |
| Demirkapu H *et al*, 2023 | Yes | Yes | Yes | Yes | Yes | Yes | Yes |
| Nimmons D *et al,* 2023 | Yes | Yes | Yes | Yes | Yes | Yes | Yes |
| Finkelstein A *et al*, 2023^80^ | Yes | Yes | Yes | Yes | Yes | Yes | Yes |
| De Vleminck A *et al*, 2023^48^ | Yes | Yes | Yes | Yes | Yes | Yes | Yes |
| Smith KM *et al*, 2022^36^ | Yes | Yes | Yes | Yes | Yes | Yes | Yes |
| Xu L *et al,* 2022^38^ | Yes | Yes | Yes | Yes | Yes | Yes | Yes |
| Carter C *et al*, 2022^72^ | Yes | Yes | Yes | Yes | Yes | Yes | Yes |
| Glaudemans J *et al*, 2020^68^ | Yes | Yes | Yes | Yes | Yes | Yes | Yes |
| Kendell C *et al*, 2020^73^ | Yes | Yes | Yes | Yes | Yes | Yes | Yes |
| Suen L *et al*, 2020^42^ | Yes | Yes | Yes | Yes | Can’t tell | Yes | Yes |
| Abu Al Hamayel *N et al*, 2019^39^ | Yes | Yes | Yes | Yes | Yes | Yes | Yes |
| Miller H *et al*, 2019^77^ | Yes | Yes | Yes | Yes | Yes | Yes | Yes |
| Tilburgs B *et al*, 2018^69^ | Yes | Yes | Yes | Yes | Yes | *No* | Yes |
| Reich A *et al*, 2019^41^ | Yes | Yes | Yes | Yes | Yes | Yes | Yes |
| Bollig G *et al*, 2016^79^ | Yes | Yes | Yes | Yes | Yes | Yes | Yes |
| Bernard C *et al*, 2020^74^ | Yes | Yes | Yes | Yes | Yes | Yes | Yes |
| **First author and year** | **Screening questions** | | **Quantitative descriptive studies** | | | | |
|  | **S1. Are there clear research questions?** | **S2. Do the collected data allow to address the research questions?** | **4.1. Is the sampling strategy relevant to address the research question?** | **4.2. Is the sample representative of the target population?** | **4.3. Are the measurements appropriate?** | **4.4. Is the risk of nonresponse bias low?** | **4.5. Is the statistical analysis appropriate to answer the research question?** |
| Caplan H *et al*, 2023 | Yes | Yes | Yes | Yes | Yes | Yes | Yes |
| Stevens J *et al*, 2023^47^ | Yes | Yes | Yes | Yes | Yes | Yes | Yes |
| Hayashi S *et al,* 2023^61^ | Yes | Yes | Yes | Yes | Yes | Can’t tell | No |
| Bzura M *et al*, 2022 | Yes | Yes | Yes | Yes | Can’t tell | No | Yes |
| McLarty S *et al*, 2022^37^ | Yes | Yes | Yes | Yes | Can’t tell | Can’t tell | Yes |
| Whyte S *et al*, 2022^76^ | Yes | Yes | Can’t tell | Can’t tell | Yes | Can’t tell | Yes |
| Busa C *et al*, 2022^81^ | Yes | Yes | Yes | Yes | Yes | Can’t tell | Yes |
| Scholten *G et al,* 2018^49^ | Yes | Yes | Yes | Yes | Yes | Can’t tell | Yes |
| Luck T et al, 2017^82^ | Yes | Yes | Yes | Yes | Yes | Yes | Yes |
| Aoki T *et al*, 2017^63^ | Yes | Yes | Yes | Can’t tell | Yes | Can’t tell | Yes |
| Lim MK *et al*, 2022^83^ | Yes | Yes | Yes | Yes | Yes | Yes | Yes |
| Hamada S *et al*, 2019^64^ | Yes | Yes | Yes | Yes | Yes | Yes | Yes |
| Musa I *et al*, 2015^59^ | Yes | Yes | Yes | Can’t tell | Yes | No | Yes |
| **First author and year** | **Screening questions** | | **Randomised controlled trials** | | | | |
|  | **S1. Are there clear research questions?** | **S2. Do the collected data allow to address the research questions?** | **2.1. Is randomization appropriately performed?** | **2.2. Are the groups comparable at baseline?** | **2.3. Are there complete outcome data?** | **2.4. Are outcome assessors blinded to the intervention provided?** | **2.5 Did the participants adhere to the assigned intervention?** |
| Yoshihara-Kurihara H *et al*, 2024 | Yes | Yes | Can’t tell | Yes | Yes | Yes | Yes |
| **First author and year** | **Screening questions** | | **Non-randomised studies** | | | | |
|  | **S1. Are there clear research questions?** | **S2. Do the collected data allow to address the research questions?** | **3.1. Are the participants representative of the target population?** | **3.2. Are measurements appropriate regarding both the outcome and intervention (or exposure)?** | **3.3. Are there complete outcome data?** | **3.4. Are the confounders accounted for in the design and analysis?** | **3.5. During the study period, is the intervention administered (or exposure occurred) as intended?** |
| Van der Plas A *et al*, 2022^67^ | Yes | Yes | Yes | Yes | Yes | Yes | Yes |
| **First author and year** | **Screening questions** | | **Mixed methods studies** | | | | |
|  | **S1. Are there clear research questions?** | **S2. Do the collected data allow to address the research questions?** | **5.1. Is there an adequate rationale for using a mixed methods design to address the research question?** | **5.2. Are the different components of the study effectively integrated to answer the research question?** | **5.3. Are the outputs of the integration of qualitative and quantitative components adequately interpreted?** | **5.4. Are divergences and inconsistencies between quantitative and qualitative results adequately addressed?** | **5.5. Do the different components of the study adhere to the quality criteria of each tradition of the methods involved?** |
| Ferguson C M *et al*, 2024 | Yes | Yes | Yes | Yes | No | Yes | Yes |
| Stevens J, *et al*, 2024 | Yes | Yes | Yes | Yes | Yes | Yes | Yes |
| Winnifrith T *et al*, 2024 | Yes | Yes | Yes | Yes | Yes | Can’t tell | No |
| Tietbohl C K *et al*, 2024 | Yes | Yes | Yes | Yes | Yes | Yes | No |
| Gardener AC *et al*, 2022^84^ | Yes | Yes | Yes | Yes | Yes | Yes | Yes |
| Ohnuki Y *et al*, 2022^62^ | Yes | Yes | No | Yes | Yes | Yes | No |
| Canny A *et aI,* 2022 | Yes | Yes | Yes | Yes | Yes | Yes | Yes |
| Tsuda S *et al*, 2020 | Yes | Yes | Yes | Yes | Yes | Yes | Yes |
| De Vleminck A *et al*, 2018^50^ | Yes | Yes | Yes | Yes | Yes | Yes | Yes |
| Lum H *et al*, 2017^40^ | Yes | Yes | Yes | No | No | Yes | Yes |
| Van Wijmen M *et al*, 2014^70^ | Yes | Yes | Yes | Yes | Yes | Yes | Can’t tell |
