# Supplementary material for: Patient perceptions of advance care planning within primary care: a systematic review of facilitators and barriers
Source: BMC Prim Care. 2025 Oct 31;26:337. doi: 10.1186/s12875-025-03028-0 (PMC12577347; doi:10.1186/s12875-025-03028-0)
Supplement: Supplementary file 4 — Additional file 4. [file 12875_2025_3028_MOESM4_ESM.docx]

**Additional file 4** Examples of raw data and qualitised data from included studies

| **Study** | **Raw data** | **Qualitised data** |
| --- | --- | --- |
| Musa I *et al*, 2015^59^ | “Of the 1,823 respondents, 796 (44%) replied that if they were unable to express themselves, they would leave decisions about their health to others. Of these, 603 (76%) agreed with the statement that they would trust their doctor/health professionals to make these decisions” | Barriers;  Professional Factors;  Patients trust doctors to make right decisions for them. |
| Luck T *et al*, 2017^82^ | “The most frequently stated reasons for not having ADs were that the older adults trust their relatives or physicians to make the right decisions for them when necessary (stated by 59.4% and 44.8% of those without ADs).” |  |
| Busa C *et al*, 2022^81^ | “A third (34%) of participants with higher education considered EOL conversations important, while this proportion among participants with secondary or primary education was lower (22% and 23%). (P value <0.001)” | Facilitators;  Personal characteristics;  Educated to University level |
| Whyte S *et al*, 2022^76^ | “Participants who exhibit the illusion of control bias are more likely to prefer the initial discussion of ACP to happen in earlier life stages [difference by 2.5 years (p=0.005) for Australian general public participants and 7.6 years (p=0.035) for health professionals, respectively]” | Barriers;  Features of the ACP conversation;  Correct timing of the conversations (may be early or later, depending on the individual) |
