# Supplementary material for: Patient perceptions of advance care planning within primary care: a systematic review of facilitators and barriers
Source: BMC Prim Care. 2025 Oct 31;26:337. doi: 10.1186/s12875-025-03028-0 (PMC12577347; doi:10.1186/s12875-025-03028-0)
Supplement: Supplementary file 5 — Additional file 5. [file 12875_2025_3028_MOESM5_ESM.docx]

**Additional file 5** Example excerpts from selected articles, mapped to themes

|  | **Barriers** | **Facilitators** |
| --- | --- | --- |
| **Professional Factors** | *Relationship with GP*   - ‘It might be inappropriate to bring up such personal issues in a doctor’s appointment’^36^ - ‘Not knowing if his [GP] beliefs and values are the same as mine’^36^ - ‘Those [ACP] agreements will not be kept.’^34^   *GP skills and attributes*   - ‘Family doctor would not listen’^32^ - ‘Understanding all the medical terminology that’s being thrown at you instead of just an old-fashioned country doctor approach where they … and tell you in layman’s terms’^49^ - ‘Some doctors may be very uncomfortable with [the conversation] and do a poor job of it’^28^   *Role of GP in ACP*   - ‘My physicians will make the right decisions for me’^44^ - ‘I just trust in the ones who care for me. What they think is best.’^51^ | *Relationship with GP*   - ‘I can say things to [Dr. S] that I might be hesitant to say to someone I don't know that well.’^28^ - ‘I think you have to have such a good relationship with your doctor before you have the conversation, and I think it only makes it just better.’^49^ - ‘Regular appointments with GP facilitates ACP’^40^   *GP skills and attributes*   - ‘They [GP] are being professional, but sometimes there was a gentleness and caring that was in her voice. … ‘I’m really listening. I am respecting’. …and if you can convey that in your voice and eye contact, I think it will ease the conversation’^49^ - ‘Characteristics such as empathy, tolerance, and being a people person … (improved trust)’^24^   *Role of GP in ACP*   - ‘72% of participants answered ‘physicians’ … whose task it was to talk with the patients about their choices.’^33^ - ‘I think doctors should be fully able to discuss all alternatives and all outcomes with the patient and be realistic’^38^ - ‘It was really him [GP] pushing these forms at us that we finally took action’^37^ |
| **Patient Factors** | *Perceptions of Self*   - ‘I believe I can be cured from my disease’^32^ - ‘I feel that I am too young for making those types of decisions’^36^ - ‘I’m not interested in this at all. [...] It’s just, I’m still too active, you know?’^34^   *Role of Family*   - ‘She [referring to her daughter] doesn’t want to hear that mom could die and she said, ‘I don’t want to hear about you dying, mama.’^38^ - ‘I don’t want arguments arising’^36^ - ‘I will follow my family’s opinion.’^21^   *Personal goals and preferences*   - *­*‘[There are] such moments and such moments [You cling to life] … until the last minute’^22^ - ‘What happens will happen anyway and I’ll deal with it when it comes.’^30^ - ‘I feel that talking about death can bring death closer’^50^   *Personal Views of ACP*   - ‘The topic ADs is too complicated for me.’^44^ - ‘[I’m]too preoccupied’^36^ - ‘Thinking about the end of life makes them anxious’^42^   *Religious Influence*   - ‘I have got no power over the death, it’s all in God’s hands’^46^ - ‘Religion did not allow the drawing of an advance directive’^41^ | *Perceptions of self*   - ‘I worry about my quality of life in the future’^50^ - ‘I’d rather deal with it now when I have all my faculties than later when I might be in distress and … maybe not pick the right result’^38^   *Role of Family*   - ‘I think he [father in law, spent the end of his life in hospital being treated against his will] died about that time […] and that I said at that moment: now I am going to complete that thing [AD]’^47^ - ‘When asked who initiated the conversation to establish an advance directive, the most frequently reported response was family/friends’^25^   *Personal goals and preferences*   - ‘I don’t want to suffer’^42^ - ‘Will give an instruction that the quality of life is preferred’^22^ - ‘It gave me my independence and respect and dignity as a human being that I did have a voice in the treatment towards the end of life care’^34^   *Personal views of ACP*   - ‘It can only be done through advertising and a face-to-face meeting with a person who has signed and can explain why’^22^ - ‘It [the leaflet] gave me the final push I needed’^22^   *Personal Characteristics*   - ­‘I did a lot of terminal care and also saw a lot of people dying of cancer. … And, then I got cancer myself … and then I drew up a euthanasia directive.^47^ - ‘Those participants who found a discussion on EOL care ‘very important’, the mean age (46.97 years) .. were also significantly lower than the sample means (47.51 years)’^33^ |
| **Features of the ACP Conversation** | - ‘The forms are too long and incomprehensible’^22^ - ‘Because of limited time only medical subjects are discussed’^40^ | - ‘A separate visit should be dedicated to this conversation alone’^35^ - ‘Mailing process allows patients and family to think about their needs before the visit, improving readiness’^24^ - ‘Sometimes it’s just a nudging reminder’^37^ |
